# Supplementary material for: HIVprotI: an integrated web based platform for prediction and design of HIV proteins inhibitors
Source: J Cheminform. 2018 Mar 9;10:12. doi: 10.1186/s13321-018-0266-y (PMC5845081; doi:10.1186/s13321-018-0266-y)
Supplement: Supplementary file 2 — Additional file 2. Source code of HIVProtI web server. [file 13321_2018_266_MOESM2_ESM.zip › HIVprotI_Source-code/hivprotI/weka-3-6-6/doc/weka/core/matrix/DoubleVector.html]

DoubleVector


---


|  |  |  |
| --- | --- | --- |
| **Overview** \| **Package** \| **Class** \| **Tree** \| **Deprecated** \| **Index** \| **Help** \| **Weka's home** | |  |
| **PREV CLASS**   **NEXT CLASS** | **FRAMES**    **NO FRAMES**     **All Classes** |  |
| SUMMARY: NESTED \| FIELD \| CONSTR \| METHOD | DETAIL: FIELD \| CONSTR \| METHOD |  |


---


## weka.core.matrix Class DoubleVector

```
java.lang.Object
  weka.core.matrix.DoubleVector
```

**All Implemented Interfaces:**: java.lang.Cloneable, RevisionHandler

---

``` public class DoubleVector extends java.lang.Object implements java.lang.Cloneable, RevisionHandler ```

A vector specialized on doubles.

**Version:**
:   $Revision: 1.4 $

**Author:**
:   Yong Wang

---

**Constructor Summary**

`DoubleVector()`
  
          Constructs a null vector.

`DoubleVector(double[] v)`
  
          Constructs a vector directly from a double array

`DoubleVector(int n)`
  
          Constructs an n-vector of zeros.

`DoubleVector(int n,
double s)`
  
          Constructs a constant n-vector.


| **Method Summary** | |
| --- | --- |
| `void` | `addElement(double x)`             Adds an element into the vector |
| `int` | `capacity()`             Gets the capacity of the vector. |
| `DoubleVector` | `cat(DoubleVector v)`             Combine two vectors together |
| `java.lang.Object` | `clone()`             Clones the DoubleVector object. |
| `DoubleVector` | `copy()`             Makes a deep copy of the vector |
| `DoubleVector` | `cumulate()`             Returns a vector that stores the cumulated values of the original vector |
| `DoubleVector` | `cumulateInPlace()`             Cumulates the original vector in place |
| `DoubleVector` | `dividedBy(DoubleVector v)`             Divided by another DoubleVector element by element |
| `DoubleVector` | `dividedByEquals(DoubleVector v)`             Divided by another DoubleVector element by element in place |
| `double` | `get(int i)`             Gets a single element. |
| `double[]` | `getArray()`             Access the internal one-dimensional array. |
| `double[]` | `getArrayCopy()`             Returns a copy of the DoubleVector usng a double array. |
| `java.lang.String` | `getRevision()`             Returns the revision string. |
| `int` | `indexOfMax()`             Returns the index of the maximum. |
| `double` | `innerProduct(DoubleVector v)`             Returns the inner product of two DoubleVectors |
| `boolean` | `isEmpty()`             Checks if it is an empty vector |
| `static void` | `main(java.lang.String[] args)` |
| `DoubleVector` | `map(java.lang.String className, java.lang.String method)`             Applies a method to the vector |
| `double` | `max()`             Returns the maximum value of all elements |
| `DoubleVector` | `minus(double x)`             Subtracts a value |
| `DoubleVector` | `minus(DoubleVector v)`             Subtracts another DoubleVector element by element |
| `DoubleVector` | `minusEquals(double x)`             Subtracts a value in place |
| `DoubleVector` | `minusEquals(DoubleVector v)`             Subtracts another DoubleVector element by element in place |
| `double` | `norm1()`             Returns the L1-norm of the vector |
| `double` | `norm2()`             Returns the L2-norm of the vector |
| `DoubleVector` | `plus(double x)`             Adds a value to all the elements |
| `DoubleVector` | `plus(DoubleVector v)`             Adds another vector element by element |
| `DoubleVector` | `plusEquals(double x)`             Adds a value to all the elements in place |
| `DoubleVector` | `plusEquals(DoubleVector v)`             Adds another vector in place element by element |
| `static DoubleVector` | `random(int n)`             Returns a random vector of uniform distribution |
| `DoubleVector` | `rev()`             Returns the reverse vector |
| `void` | `set(double s)`             Set all elements to a value |
| `void` | `set(DoubleVector v)`             Set the elements using a DoubleVector |
| `void` | `set(int i, double s)`             Set a single element. |
| `void` | `set(int i0, int i1, double s)`             Set some elements to a value |
| `void` | `set(int i0, int i1, double[] v, int j0)`             Set some elements using a 2-D array |
| `void` | `set(int i0, int i1, DoubleVector v, int j0)`             Set some elements using a DoubleVector. |
| `void` | `setCapacity(int n)`             Sets the capacity of the vector |
| `void` | `setPlus(int i, double s)`             Adds a value to an element |
| `void` | `setSize(int m)`             Sets the size of the vector |
| `void` | `setTimes(int i, double s)`             Multiplies a value to an element |
| `DoubleVector` | `sign()`             Returns the signs of all elements in terms of -1, 0 and +1. |
| `int` | `size()`             Gets the size of the vector. |
| `void` | `sort()`             Sorts the array in place |
| `IntVector` | `sortWithIndex()`             Sorts the array in place with index returned |
| `void` | `sortWithIndex(int xi, int xj, IntVector index)`             Sorts the array in place with index changed |
| `DoubleVector` | `sqrt()`             Returns the square-root of all the elements in the vector |
| `DoubleVector` | `square()`             Returns the squared vector |
| `DoubleVector` | `subvector(int i0, int i1)`             Returns a subvector. |
| `DoubleVector` | `subvector(IntVector index)`             Returns a subvector. |
| `double` | `sum()`             Returns the sum of all elements in the vector. |
| `double` | `sum2()`             Returns the squared sum of all elements in the vector. |
| `double` | `sum2(DoubleVector v)`             Returns \|\|u-v\|\|^2 |
| `void` | `swap(int i, int j)`             Swaps the values stored at i and j |
| `DoubleVector` | `times(double s)`             Multiplies a scalar |
| `DoubleVector` | `times(DoubleVector v)`             Multiplies another DoubleVector element by element |
| `DoubleVector` | `timesEquals(double s)`             Multiply a vector by a scalar in place, u = s \* u |
| `DoubleVector` | `timesEquals(DoubleVector v)`             Multiplies another DoubleVector element by element in place |
| `java.lang.String` | `toString()`             Convert the DoubleVecor to a string |
| `java.lang.String` | `toString(int digits, boolean trailing)`             Convert the DoubleVecor to a string |
| `DoubleVector` | `unpivoting(IntVector index, int length)`             Returns a vector from the pivoting indices. |
| `boolean` | `unsorted()`             Returns true if vector not sorted |

**Methods inherited from class java.lang.Object**

`equals, getClass, hashCode, notify, notifyAll, wait, wait, wait`

**Constructor Detail**

### DoubleVector

```
public DoubleVector()
```

:   Constructs a null vector.

---


### DoubleVector

```
public DoubleVector(int n)
```

:   Constructs an n-vector of zeros.

    **Parameters:**: `n` - length.

---


### DoubleVector

```
public DoubleVector(int n,
                    double s)
```

:   Constructs a constant n-vector.

    **Parameters:**: `n` - length.: `s` - the scalar value used to fill the vector

---


### DoubleVector

```
public DoubleVector(double[] v)
```

:   Constructs a vector directly from a double array

    **Parameters:**: `v` - the array


**Method Detail**

### set

```
public void set(int i,
                double s)
```

:   Set a single element.

    :   **Parameters:**: `i` - Index.: `s` - a[i].

---


### set

```
public void set(double s)
```

:   Set all elements to a value

    :   **Parameters:**: `s` - the value

---


### set

```
public void set(int i0,
                int i1,
                double s)
```

:   Set some elements to a value

    :   **Parameters:**: `i0` - the index of the first element: `i1` - the index of the second element: `s` - the value

---


### set

```
public void set(int i0,
                int i1,
                double[] v,
                int j0)
```

:   Set some elements using a 2-D array

    :   **Parameters:**: `i0` - the index of the first element: `i1` - the index of the second element: `j0` - the index of the starting element in the 2-D array: `v` - the values

---


### set

```
public void set(DoubleVector v)
```

:   Set the elements using a DoubleVector

    :   **Parameters:**: `v` - the DoubleVector

---


### set

```
public void set(int i0,
                int i1,
                DoubleVector v,
                int j0)
```

:   Set some elements using a DoubleVector.

    :   **Parameters:**: `i0` - the index of the first element: `i1` - the index of the second element: `v` - the DoubleVector: `j0` - the index of the starting element in the DoubleVector

---


### getArray

```
public double[] getArray()
```

:   Access the internal one-dimensional array.

    :   **Returns:**: Pointer to the one-dimensional array of vector elements.

---


### getArrayCopy

```
public double[] getArrayCopy()
```

:   Returns a copy of the DoubleVector usng a double array.

    :   **Returns:**: the one-dimensional array.

---


### sort

```
public void sort()
```

:   Sorts the array in place

---


### sortWithIndex

```
public IntVector sortWithIndex()
```

:   Sorts the array in place with index returned

---


### sortWithIndex

```
public void sortWithIndex(int xi,
                          int xj,
                          IntVector index)
```

:   Sorts the array in place with index changed

    :   **Parameters:**: `xi` - first index: `xj` - last index: `index` - array that stores all indices

---


### size

```
public int size()
```

:   Gets the size of the vector.

    :   **Returns:**: the size

---


### setSize

```
public void setSize(int m)
```

:   Sets the size of the vector

    :   **Parameters:**: `m` - the size

---


### capacity

```
public int capacity()
```

:   Gets the capacity of the vector.

    :   **Returns:**: the capacity.

---


### setCapacity

```
public void setCapacity(int n)
```

:   Sets the capacity of the vector

    :   **Parameters:**: `n` - the capacity.

---


### get

```
public double get(int i)
```

:   Gets a single element.

    :   **Parameters:**: `i` - Index. **Returns:**: the value of the i-th element

---


### setPlus

```
public void setPlus(int i,
                    double s)
```

:   Adds a value to an element

    :   **Parameters:**: `i` - the index of the element: `s` - the value

---


### setTimes

```
public void setTimes(int i,
                     double s)
```

:   Multiplies a value to an element

    :   **Parameters:**: `i` - the index of the element: `s` - the value

---


### addElement

```
public void addElement(double x)
```

:   Adds an element into the vector

    :   **Parameters:**: `x` - the value of the new element

---


### square

```
public DoubleVector square()
```

:   Returns the squared vector

---


### sqrt

```
public DoubleVector sqrt()
```

:   Returns the square-root of all the elements in the vector

---


### copy

```
public DoubleVector copy()
```

:   Makes a deep copy of the vector

---


### clone

```
public java.lang.Object clone()
```

:   Clones the DoubleVector object.

    :   **Overrides:**: `clone` in class `java.lang.Object`

---


### innerProduct

```
public double innerProduct(DoubleVector v)
```

:   Returns the inner product of two DoubleVectors

    :   **Parameters:**: `v` - the second DoubleVector **Returns:**: the product

---


### sign

```
public DoubleVector sign()
```

:   Returns the signs of all elements in terms of -1, 0 and +1.

---


### sum

```
public double sum()
```

:   Returns the sum of all elements in the vector.

---


### sum2

```
public double sum2()
```

:   Returns the squared sum of all elements in the vector.

---


### norm1

```
public double norm1()
```

:   Returns the L1-norm of the vector

---


### norm2

```
public double norm2()
```

:   Returns the L2-norm of the vector

---


### sum2

```
public double sum2(DoubleVector v)
```

:   Returns ||u-v||^2

    :   **Parameters:**: `v` - the second vector

---


### subvector

```
public DoubleVector subvector(int i0,
                              int i1)
```

:   Returns a subvector.

    :   **Parameters:**: `i0` - the index of the first element: `i1` - the index of the last element **Returns:**: v[i0:i1]

---


### subvector

```
public DoubleVector subvector(IntVector index)
```

:   Returns a subvector.

    :   **Parameters:**: `index` - stores the indices of the needed elements **Returns:**: v[index]

---


### unpivoting

```
public DoubleVector unpivoting(IntVector index,
                               int length)
```

:   Returns a vector from the pivoting indices. Elements not indexed are
    set to zero.

    :   **Parameters:**: `index` - stores the pivoting indices: `length` - the total number of the potential elements **Returns:**: the subvector

---


### plus

```
public DoubleVector plus(double x)
```

:   Adds a value to all the elements

    :   **Parameters:**: `x` - the value

---


### plusEquals

```
public DoubleVector plusEquals(double x)
```

:   Adds a value to all the elements in place

    :   **Parameters:**: `x` - the value

---


### plus

```
public DoubleVector plus(DoubleVector v)
```

:   Adds another vector element by element

    :   **Parameters:**: `v` - the second vector

---


### plusEquals

```
public DoubleVector plusEquals(DoubleVector v)
```

:   Adds another vector in place element by element

    :   **Parameters:**: `v` - the second vector

---


### minus

```
public DoubleVector minus(double x)
```

:   Subtracts a value

    :   **Parameters:**: `x` - the value

---


### minusEquals

```
public DoubleVector minusEquals(double x)
```

:   Subtracts a value in place

    :   **Parameters:**: `x` - the value

---


### minus

```
public DoubleVector minus(DoubleVector v)
```

:   Subtracts another DoubleVector element by element

    :   **Parameters:**: `v` - the second DoubleVector

---


### minusEquals

```
public DoubleVector minusEquals(DoubleVector v)
```

:   Subtracts another DoubleVector element by element in place

    :   **Parameters:**: `v` - the second DoubleVector

---


### times

```
public DoubleVector times(double s)
```

:   Multiplies a scalar

    :   **Parameters:**: `s` - scalar **Returns:**: s \* v

---


### timesEquals

```
public DoubleVector timesEquals(double s)
```

:   Multiply a vector by a scalar in place, u = s \* u

    :   **Parameters:**: `s` - scalar **Returns:**: replace u by s \* u

---


### times

```
public DoubleVector times(DoubleVector v)
```

:   Multiplies another DoubleVector element by element

    :   **Parameters:**: `v` - the second DoubleVector

---


### timesEquals

```
public DoubleVector timesEquals(DoubleVector v)
```

:   Multiplies another DoubleVector element by element in place

    :   **Parameters:**: `v` - the second DoubleVector

---


### dividedBy

```
public DoubleVector dividedBy(DoubleVector v)
```

:   Divided by another DoubleVector element by element

    :   **Parameters:**: `v` - the second DoubleVector

---


### dividedByEquals

```
public DoubleVector dividedByEquals(DoubleVector v)
```

:   Divided by another DoubleVector element by element in place

    :   **Parameters:**: `v` - the second DoubleVector

---


### isEmpty

```
public boolean isEmpty()
```

:   Checks if it is an empty vector

---


### cumulate

```
public DoubleVector cumulate()
```

:   Returns a vector that stores the cumulated values of the original
    vector

---


### cumulateInPlace

```
public DoubleVector cumulateInPlace()
```

:   Cumulates the original vector in place

---


### indexOfMax

```
public int indexOfMax()
```

:   Returns the index of the maximum.

    If multiple maximums exist, the index of the first is returned.

---


### unsorted

```
public boolean unsorted()
```

:   Returns true if vector not sorted

---


### cat

```
public DoubleVector cat(DoubleVector v)
```

:   Combine two vectors together

    :   **Parameters:**: `v` - the second vector

---


### swap

```
public void swap(int i,
                 int j)
```

:   Swaps the values stored at i and j

    :   **Parameters:**: `i` - the index i: `j` - the index j

---


### max

```
public double max()
```

:   Returns the maximum value of all elements

---


### map

```
public DoubleVector map(java.lang.String className,
                        java.lang.String method)
```

:   Applies a method to the vector

    :   **Parameters:**: `className` - the class name: `method` - the method

---


### rev

```
public DoubleVector rev()
```

:   Returns the reverse vector

---


### random

```
public static DoubleVector random(int n)
```

:   Returns a random vector of uniform distribution

    :   **Parameters:**: `n` - the size of the vector

---


### toString

```
public java.lang.String toString()
```

:   Convert the DoubleVecor to a string

    :   **Overrides:**: `toString` in class `java.lang.Object`

---


### toString

```
public java.lang.String toString(int digits,
                                 boolean trailing)
```

:   Convert the DoubleVecor to a string

    :   **Parameters:**: `digits` - the number of digits after decimal point: `trailing` - true if trailing zeros are to be shown

---


### getRevision

```
public java.lang.String getRevision()
```

:   Returns the revision string.

    :   **Specified by:**: `getRevision` in interface `RevisionHandler`
    :   **Returns:**: the revision

---


### main

```
public static void main(java.lang.String[] args)
```


---


|  |  |  |
| --- | --- | --- |
| **Overview** \| **Package** \| **Class** \| **Tree** \| **Deprecated** \| **Index** \| **Help** \| **Weka's home** | |  |
| **PREV CLASS**   **NEXT CLASS** | **FRAMES**    **NO FRAMES**     **All Classes** |  |
| SUMMARY: NESTED \| FIELD \| CONSTR \| METHOD | DETAIL: FIELD \| CONSTR \| METHOD |  |


---
